# A unique gating mechanism revealed by the cryo-EM structure of monomeric ATP9A flippase

**DOI:** 10.1016/j.jbc.2025.110631

**Published:** 2025-08-26

**Authors:** Kazuhiro Abe, Parthiban Marimuthu, Yuheng Qian, Chai C. Gopalasingam, Christoph Gerle, Hideki Shigematsu, Kotaro Tanaka, Himanshu Khandelia

**Affiliations:** 1 Department of Chemistry, Faculty of Science, Hokkaido University, Sapporo, Japan; 2 Pharmaceutical Science Laboratory (PSL – Pharmacy) and Structural Bioinformatics Laboratory (SBL – Biochemistry), Faculty of Science and Engineering, Åbo Akademi University, Turku, Finland; 3 Graduate School of Pharmaceutical Sciences, Nagoya University, Nagoya, Japan; 4 RIKEN SPring-8 Center, Hyogo, Japan; 5 Japan Synchrotron Radiation Research Institute (JASRI), SPring-8, Hyogo, Japan; 6 Department of Physics Chemistry and Pharmacy, University of Southern Denmark, Odense, Denmark

**Keywords:** ATPase, cryo-EM, lipid transport, membrane protein, phospholipid, transporter, molecular dynamics simulations

## Abstract

Among mammalian P4-ATPase flippases, only ATP9A and ATP9B do not require the auxiliary subunit CDC50 protein. Whilst its yeast homolog, Neo1, is essential for cell survival, little is known about mammalian ATP9A. We present cryo-EM structures of human monomeric ATP9A at a resolution reaching 2.2 Å, in the outward-facing E2P state. Two distinguishable conformations were obtained from a single sample, one with its outward gate open and the other in its closed form. Unlike canonical gating observed for most P-type ATPases, which is driven by the movement of transmembrane (TM) helices 1 and 2 linked to the A domain, outward gating in ATP9A is achieved by the movement of TM6–10 helices, likely initiated by the unwinding of TM6. As a result, the volume of the phospholipid binding cavity in the open state surpasses that of other flippases, which could allow binding of phospholipids with larger hydrophilic headgroups than that of phosphatidylserine. ATP9A shows an ATPase activity that is significantly increased by the addition of phospholipids that retain the overall negative charge, including phosphatidylserine, phosphatidylinositol, and its phosphorylated species, compared with other electroneutral phospholipids. The observation of spontaneous binding of phosphorylated species of phosphatidylinositol in molecular simulation reinforces this fact. Our data provide mechanistic rationales for ATP9A gating, achieved by the rearrangement of the second half of the TM helices. Since TM4–TM10 is anchored by the CDC50 protein subunit in other flippases, the here-observed outward gating mechanism is unique to P4B-type flippases, which function as a monomer.

P-type ATPases are a large membrane protein family that mediate ATP-driven uphill translocation of their substrate across the membrane (1). Different from cation-transporting P2-type ATPases, P4-type ATPases act as a flippase that translocates phospholipid from the exofacial to the cytofacial leaflet of the lipid bilayer to keep an asymmetric distribution of the phospholipids in the biological membrane (2), which, together with phospholipid scramblases, have been implicated in many cellular processes, including blood clotting, membrane traffic, and apoptosis (3). P4-ATPases are classified into three subgroups, called P4A, P4B, and P4C types. In humans, the P4A type requires an auxiliary subunit CDC50 protein for functional expression in the cell. Among all 14 members of the P4-ATPases, only ATP9A and ATP9B, belonging to the P4B type, do not require CDC50 protein, thus functioning as a single catalytic subunit.

Like other P-type ATPases, the transport mechanisms of P4-ATPases are described as a cyclic conversion of the enzyme conformations, E1, E2, and their autophosphorylated forms, E1P and E2P, according to the Post-Albers type reaction scheme (4, 5, 6). The overall fold of P4-ATPases also resembles those of well-studied P2-type ATPases (7, 8). The catalytic subunit consists of 10 transmembrane (TM) helices in which the phospholipid-binding site is located and three cytoplasmic domains involved in ATP hydrolysis. ATP is bound to the nucleotide-binding (N) domain, and its terminal phosphate is transferred to the catalytic aspartate residue in the invariant DKTGT sequence located at the phosphorylation (P) domain to form a phosphoenzyme intermediate E1P state. After phosphorylation, the DGET loop in the actuator (A) domain moves close to the P-domain and covers the aspartylphosphate in the E2P state. Connection of the A domain to TM1 and TM2 allows rearrangement of TM helices *via* A domain movement and, as a consequence, induces exoplasmic gate opening to incorporate phospholipids from the outer leaflet. Subsequently, exoplasmic gate closure is induced by the phospholipid occlusion, in which a polar headgroup of the specific phospholipid is accommodated while leaving hydrophobic acyl chains dissolved in the lipid bulk of the surrounding membrane. This local conformational change occurring in TM1 and TM2 is transmitted to the A domain and expedites E2P dephosphorylation, which is usually described as E2-P_i_ transition state. The aforementioned series of conformational changes are commonly observed in hATP8A1 (8), hATP11C (9, 10), and yeast Drs2 (7), all of which form a heterodimer with the auxiliary subunit, CDC50 protein.

The ATP9A yeast homolog, Neo1 (11), is responsible for neomycin resistance and has also been demonstrated to be critical for cell survival (12). In humans, it was shown that ATP9A is involved in the process of insulin secretion (13), exosome recycling (14), and the exosome release pathway (15). Recently, it has been reported that the nonsense mutations of ATP9A cause autosomal recessive hypotonia, intellectual disability, and attention deficit hyperactivity disorder because of abnormal endosomal recycling (16). Despite an accumulation of knowledge of the physiological importance of ATP9A in humans, little is known about its molecular mechanism. To address this, we solved cryo-EM structures of human ATP9A in the outward-facing E2P state. Unexpectedly, we found two distinct conformations existing in the equilibrium: one with a large cavity at the phospholipid-binding site and the other in a closed form. Together with the functional ATPase measurements and molecular dynamics (MD) simulations, our data revealed that an unusually large and positively charged phospholipid-binding site is formed by the unique conformational change in ATP9A, suggesting that ATP9A acts as a nonspecific flippase with a preference for net-negative charged phospholipids.

## Results and discussion

### Cryo-EM analysis of human ATP9A

**Figure 1:**
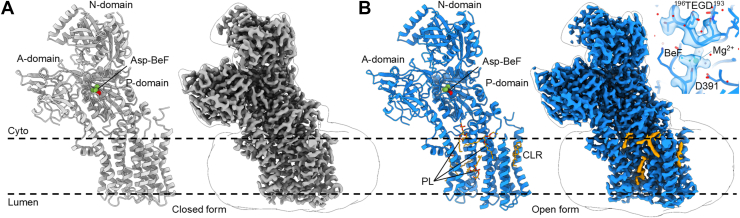
**Cryo-EM structures of closed and open forms of human ATP9A in E2P state.** Overall structures in the closed (*A*, *gray cartoon and surface*, PDB ID: 9VDL) and open (*B*, *blue cartoon and surface*, PDB ID: 9VDK) forms found in the BeF-bound E2P state viewed from the membrane plane (*dotted lines*) with cytoplasmic side up. Transparent surfaces represent Gaussian-filtered low-contoured maps showing molecular envelopes and micelles. A-, P-, and N-domains are indicated. BeF-bound catalytic Asp391 are shown as *green spheres*. Bound phospholipids (PLs) and cholesterol (CLR) in the open form are indicated with *yellow sticks and surface*. *Inset* in *B* represents a close-up of the phosphorylation site showing the density map (transparent surface) within 4 Å of Asp391, BeF, and the DGET loop from the A-domain. BeF, beryllium fluoride; PDB, Protein Data Bank.

To obtain molecular insights into the transport mechanism of ATP9A flippase, human ATP9A was overexpressed in the human embryonic kidney 293 cells (17, 18), purified in the presence of the phosphate analog, beryllium fluoride (BeF) (Fig. S1) (19), and then subjected to cryo-EM structure determination 1(Figs. 1 and S2, Table S1). The overall structure shows a typical fold seen in the catalytic subunit of other P4A-ATPases (10) as well as in the yeast homolog Neo1 (11). The phosphate analog BeF is bound to the catalytic Asp391 located in the ^391^DKTG motif of the P domain and is shielded from bulk water by being covered by the ^193^DGET loop in the A domain (Fig. 1*B*, *inset*), indicating that the conformation of the enzyme is fixed in the E2P state (10, 20). To our surprise, however, the cryo-EM classification analysis revealed the presence of two distinguishable conformations in a single image dataset obtained from the same sample (Figs. S2 and S3), which we termed the closed (Fig. 1*A*) and open forms (Fig. 1*B*), analyzed at 2.31 Å and 2.18 Å, respectively (Movie S1). Most side chains in the TM region (except bound phospholipid as described later) are unambiguously determined, as seen in their high DAQ score (Fig. S3) (21). With the aim of obtaining structures of inward-facing E1-ATP and outward-occluded E2-P_i_ states, we also performed cryo-EM analysis for the samples purified under differing biochemical conditions, including in the presence of a nonhydrolyzable ATP analog (AMPPCP) and transition state analog aluminum fluoride (AlF) with phosphatidylserine (PS) added, respectively. However, all the structures obtained are almost identical. Only the open form is obtained from the AMPPCP- and AlF-added samples, with resolutions of 3.0 Å (RMSD 0.634 Å compared with the BeF open form) and 2.6 Å (RMSD 0.388 Å compared with the BeF open form), respectively (Fig. S4). We could not find any significant difference in either the relative orientation of the cytoplasmic domains, TM helices, or EM density at the phospholipid-binding site. Despite the absence of externally added phosphate, we observed clear density for phosphate binding to the catalytic Asp391 even in the presence of 5 mM AMPPCP (Fig. S4, *inset*). It is most likely that cellular phosphate remains firmly bound to the enzyme throughout the purification and cryo-grid preparation process. These results suggest that ATP9A shows a strong preference for the E2P state, which may be one of the reasons for its slow turnover as described later.

### Open and closed forms in the E2P state

**Figure 2:**
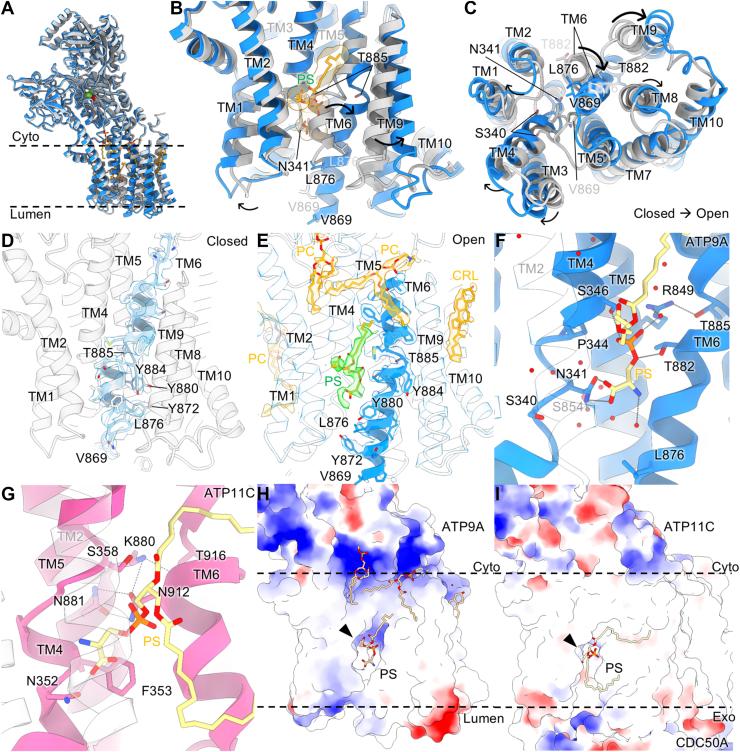
**Comparison of the molecular conformation between open and closed forms.***A*, superimposition of whole structures (*A*) of closed (*gray*, PDB ID: 9VDL) and open (*blue*, PDB ID: 9VDK) forms are shown as *cartoon representations*. *B* and *C*, close-up views of the TM helices from the parallel (*B*) or luminal side (*C*) of the membrane plane. Bound PS (*yellow*) and some of the important residues are shown as *sticks*. *Arrows* indicate the movement from closed to open forms. *D* and *E*, structure of TM6 in closed (*D*) and open (*E*) states with EM density within 4 Å from the model. EM densities for bound PS in the canonical binding site (*green surface*) and other lipids (*yellow surface*) and cholesterol (CLR) are also shown. *F*, a close-up of the canonical phospholipid-binding site in the open form is shown, viewed from the position where TM2 (shown as a transparent helix) is located. *Red spheres* represent water molecules. *Dotted lines* are connecting atoms within 3.5 Å distance. *G*, canonical phospholipid-binding site in the E2P state of ATP11C (*pink*, PDB ID: 7BSU) from the viewpoint similar to (*F*). *H* and *I*, surface representations of the TM regions in ATP9A (*H*) and the ATP11C–CDC50A complex (*I*) with their electrostatic potentials (*blue*: positive, *red*: negative). PS, phosphatidylserine; TM, transmembrane.

Comparison of open and closed conformations reveals that, while the relative orientation of cytoplasmic domains remains the same, the arrangement of TM6–TM10, especially TM6, shows a significant difference (Fig. 2, *A*–*C*). Like other P4-ATPases (8, 10) and P2-ATPases (22, 23, 24), TM6 of ATP9A is unwound in the middle of the helix. In the closed form of ATP9A, the luminal half of TM6 adopts a bent conformation and is situated in the position where the canonical phospholipid-binding site is located (Fig. 2*D*). In marked contrast, the luminal half of TM6 takes up a relatively straighter conformation in the open form (Fig. 2*E*). The displacement of the TM6 between the closed and open state results in more than 10 Å displacement in the largest case. Due to the TM6 conformational difference, there is a space between TM2 and TM6 of the open form, in which we observed phospholipid-like EM density (Fig. 2*E*). Although the EM density indicates the presence of two acyl chains, the headgroup density is not defined well enough to allow a clear molecular identification (Fig. S3). Still, since the dimension of the binding pocket is large enough to accommodate a PS headgroup with several water molecules, we hypothetically modeled PS into the binding pocket (Fig. 2, *B* and *E*). The unclear positioning of the hydrophilic headgroup of PS hinders our understanding of detailed interaction. However, the PS molecule, modeled based on the stronger density for the phosphate group and the part of acyl chains predicted to be in a position where it can interact with several hydrophilic residues, including Asn341 located in the unwound part of TM4 and Ser346 in TM4 and Thr882 in TM6 (Fig. 2*F*). The predicted binding mode in ATP9A is similar to those for P4A-type flippases such as ATP11C (Fig. 2*G*) (10), except the contribution of a positively charged amino acid, Arg849, in TM5. In contrast to Lys880 in ATP11C, which stabilizes TM5 and TM6 but does not contribute to the PS binding (10), the corresponding Arg849 in ATP9A is facing the surface of the binding site and likely contributes to the PS coordination *via* water (Fig. 2*F*). Due to this structural difference, the substrate binding pocket of ATP9A shows an electrostatically positive environment, which may contribute to attracting negatively charged phospholipid headgroups (Fig. 2, *H* and *I*).

**Figure 3:**
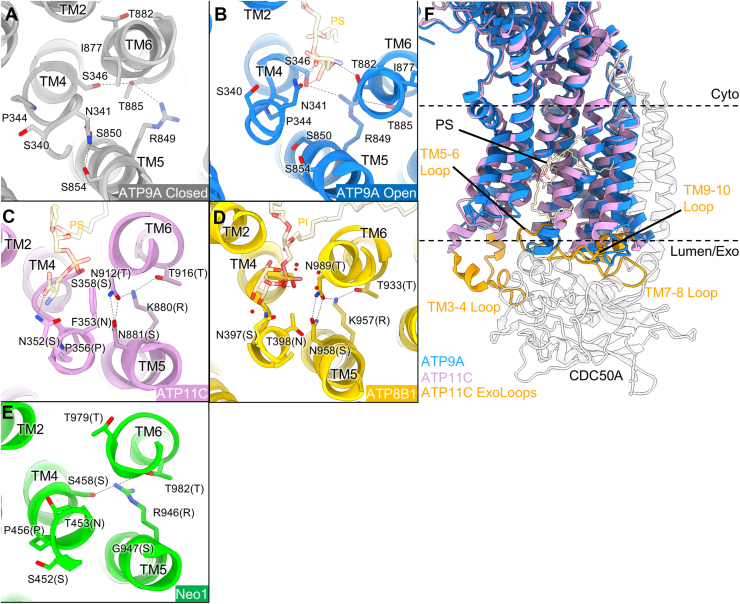
**Role of Arg849 in TM5 upon gating.***A*–*D*, close-up of phospholipid-binding site in ATP9A E2P closed form (*A*, PDB ID: 9VDL), its open form (*B*, PDB ID: 9VDK), ATP11C PS-bound E2P state (*C*, PDB ID: 7BSU), ATP8B1 PI-bound E2-Pi state (*D*, PDB ID: 8OXC) and Neo1 E2P state (*D*, PDB ID: 7RD6), viewed from the luminal/extracellular side. Some of the important residues are shown as *sticks* and indicated in the figures. In *C*–*E*, their corresponding amino acids in ATP9A are indicated in *parentheses*. *Dotted lines* are connecting atoms within 3.5 Å distance. *E*, the ATP9A (*blue ribbons*) open form is superimposed on the complex of ATP11C (*pink*) and CDC50A (*gray*). Exoplasmic loops of ATP11C, which form intimate interactions with CDC50A, are shown as *orange ribbons*. PDB, Protein Data Bank; PS, phosphatidylserine; TM, transmembrane.

Besides the contribution of Arg849 for creating a positively charged environment in the binding pocket, this residue may also be a key to stabilizing both open and closed forms (Fig. 3). Unwound TM6 of the closed form is bent in the middle of the helix and occupies the phospholipid-binding site position (Fig. 2, *B* and *D*). The hydrogen bond between Ser346 (TM4) and Thr885 (TM6) keeps these two helices in proximity to each other (Fig. 3*A*). The position of Thr885 in the closed form is likely stabilized by Arg849 from the opposite side. Because of the tight arrangement of TM4 and TM6, the steric hindrance because of the Ile877 side chain (TM6) affects the conformation of the TM4 unwinding and thus prevents the Asn341 side chain facing toward the phospholipid-binding site and makes hydrogen bonds to Ser850 and Ser854 (Fig. 3*A*). In contrast, Arg849 is located in between Ser346 and Thr885 to segregate TM4 and TM6 in open form (Fig. 3*B*). Due to TM6 rotation, Ile877 is moved farther from the canonical binding site, and Asn341 in TM4’s unwound state is facing toward the phospholipid-binding site to coordinate the hydrophilic headgroup of the phospholipid. We therefore conclude that, at least, the relative orientation of the Arg849 side chain to the TM6 contributes to forming the phospholipid-binding site.

One reason for the observed variable conformation of TM6 in ATP9A is the lack of hydrogen bonds between TM5 and TM6. Comparison with the PS flippase ATP11C (10) shows that the hydrogen bond network between Asn881 and Asn912 with Lys880 and Thr916 helps to fix the position of TM6 relative to TM5 (Fig. 3*C*). Similarly, these tight interactions between TM5 and TM6 are also observed in human ATP8B1 with phosphatidylinositol (PI) bound (Fig. 3*D*) (25). These two asparagine residues in ATP11C and ATP8B1 are replaced by shorter serine and threonine side chains in ATP9A (Fig. 3, *A* and *B*), and thus hydrogen bonds between Arg849 and Thr885 are the sole hydrophilic interactions that connect TM5 and TM6. Furthermore, another crucial difference is the interaction with CDC50 protein; ATP11C intimately interacts with CDC50A *via* its exoplasmic loops, which are absent in ATP9A (Fig. 3*F*). Because of such extensive interactions between the two subunits, an exoplasmic portion of TM helices, except TM1 and TM2 in ATP11C, scarcely moves during its transport cycle (7, 8, 10). The lack of this anchoring effect by the CDC50 subunit in the monomeric ATP9A allows the large rearrangement of TM6 and neighboring TM helices (Fig. 2, *B* and *C*). Since its yeast homolog monomeric Neo1 (11) also has an arginine (Arg946) in TM5 (Fig. 3*E*), a similar mechanism could be expected when phospholipid is accommodated in the binding pocket, although the structure of the phospholipid-bound form has yet to be solved.

**Figure 4:**
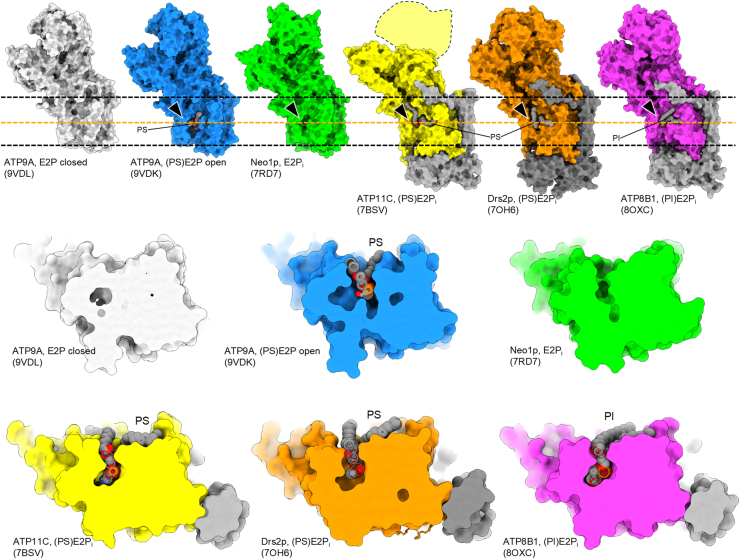
**Dimensions of the phospholipid-binding site in different flippases.** Amino acid models of indicated flippases are shown as *surface representations*. Their whole structures viewed from the membrane plane are shown in the *upper panel*. Membrane slices, viewed from the luminal/extracellular side of the membrane, at the position indicated with an *orange dotted line*, are shown in the *lower panel*. Bound phospholipids are shown as *spheres*. *Black arrowheads* indicate the phospholipid-binding site. PDB codes for each structure are indicated in the figure. PDB, Protein Data Bank.

The unusual size of the phospholipid binding pocket in the open form of ATP9A is highlighted by its comparison with those of several other P4-flippases (Fig. 4). The phospholipid-binding site is completely obscured in the closed form of ATP9A, in stark contrast to its open form. Although no phospholipid is bound, the E2P state of Neo1 shows a longitudinal cleft-like structure (11). The binding pocket of PS flippases such as human ATP11C (10), yeast Drs2p (26), or human ATP8B1 with PI bound (25) is more tightly molded to the substrate. Compared with any of the aforementioned examples, the phospholipid binding pocket in ATP9A is found to be significantly larger.

### Activation of ATP hydrolysis by negatively charged phospholipids

**Figure 5:**
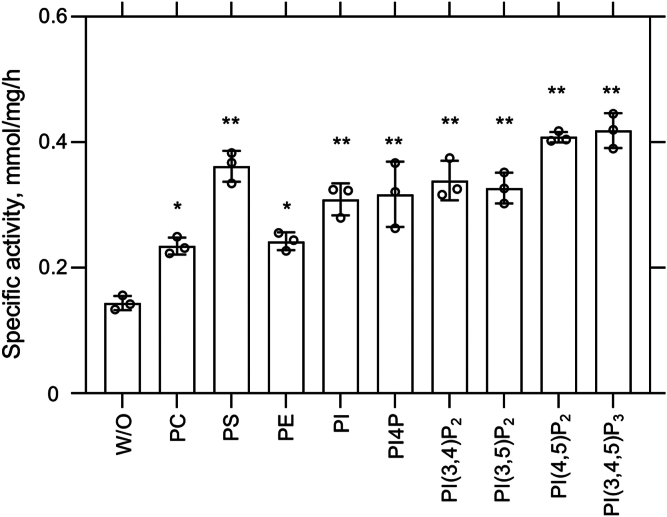
**Specific ATPase activities of purified ATP9A in the presence of phospholipids.** ATP9A is purified in the absence of phosphate analog and measured its ATPase activity in the absence (W/O) or presence of indicated phospholipids added (final concentrations of 20 μM). Background ATPase activity is subtracted by setting the BeF-inhibited sample as a blank. Data plotted are mean ± SD from three independent experiments (∗*p* < ∗0.01 and ∗∗*p* < 0.0001, one-way ANOVA, compared with ATPase activity without phospholipids, W/O). BeF, beryllium fluoride; W/O, without.

The unusually large binding pocket in ATP9A prompted us to consider the possibility that phospholipids with a hydrophilic headgroup larger than that of PS could serve as a transport substrate. According to the Post-Albers type reaction scheme, the binding and following occlusion of the transport substrate accelerates the dephosphorylation of E2P, which results in increased ATPase activity (10, 27). We measured the ATPase activity of purified ATP9A in the presence of various phospholipids as shown in Figure 5. Compared with the basal ATPase activity in the absence of phospholipid, the addition of PS, PI, and its phosphorylated forms (phosphatidylinositol phosphates [PIPs]), those with net negatively charged phospholipids showed a significant increase in its ATPase activity (Fig. 5). In contrast, net neutral phospholipids, including phosphatidylcholine and phosphatidylethanolamine, show lesser activation. These data suggest that ATP9A may work as a flippase with broad specificity but prefers negatively charged phospholipids. Our *in vitro* data are consistent with the observed large and positively charged phospholipid-binding site of ATP9A (Fig. 2*H*). As we did not add any phospholipids exogenously during the purification step, the observed phospholipid-like cryo-EM density is likely to be an average of phospholipids carried over from the cell membrane, resulting in the noisy density observed in the cryo-EM structure (Fig. 1*E*).

### Coarse-grained simulation of spontaneous phospholipid binding

**Figure 6:**
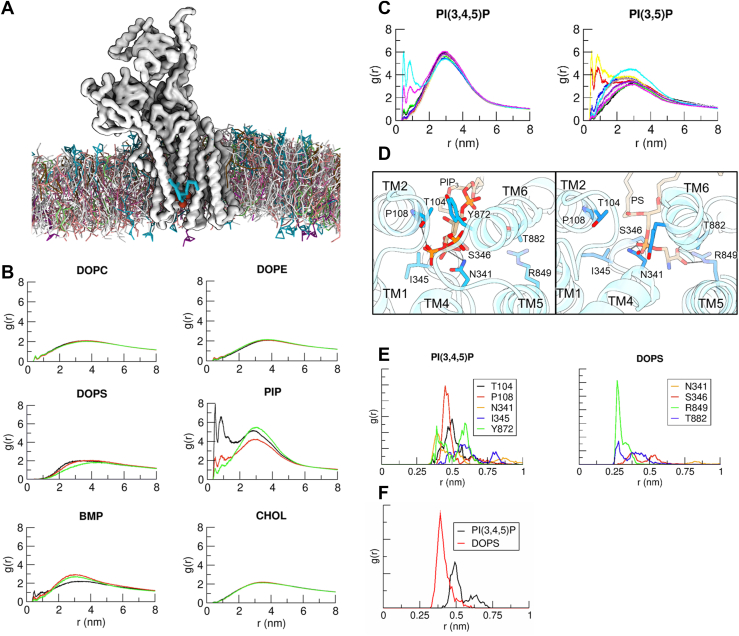
**Molecular simulations.***A*, simulation snapshot from the coarse-grained simulations showing a PI(3,5)P_2_ lipid bound to the lipid-binding site. *B*, radial distribution functions (RDFs, normalized distance histograms) between the indicated lipid headgroup and the center of mass (COM) of the binding site residues (Gln92, Leu99, Thr104, Tyr105, Ser340, Asn341, Ile345, Ser346, Arg849, Ser850, Gln857, Tyr872, and Thr882) of the protein. The *curves* correspond to simulations in the presence of PI(3,4,5)P_3_ (*black*), PI(3,5)P_2_ (*red*), and PI(4,5)P_2_ (*green*), respectively. The strongest interactions are observed for PIPs, which is the only lipid showing significant peaks at a distance of less than 1 nm, indicating strong interactions of the headgroup with the binding site. PI(3,4,5)P_3_ (*black curve*) has the strongest interactions with the binding site. Single representative simulation replicas were used for these plots. The data show that PIPs are most likely to bind to the lipid-binding site. *C*, after finding that PIPs are most likely to bind the protein, we placed both PI(3,5)P_2_ and PI(3,4,5)P_2_ in the membrane in the same simulation setup and simulated 10 replicas of this system. Each color in the panels in plot C represents the RDF for a single replica. The data show that the lipid-binding site has no specific preference for PI(3,4)P_2_ or PI(3,4,5)P_3_. See Table S2 for the lipid composition of the membranes. *D*, representative snapshots from all-atom simulation for PI(3,4,5)P_3_ (*left*) and DOPS (*right*) bound to the phospholipid-binding site, viewed from the luminal side. Potential hydrogen bonds are shown by connecting atoms within 3.5 Å distance. Amino acids within 4 Å distance from either of the phospholipids are shown as *sticks* as candidates for van der Waals interaction. *E*, RDFs between the side chains of binding site residues, which interact most with PI(3,4,5)P_3_ and DOPS in the all-atom simulations. The RDFs between indicated residues and the closest phosphate atom of the PI(3,4,5)P_3_ headgroup (*left*) or the carboxylate carbon atom of the PS headgroup (*right*) were plotted, respectively. The different colors correspond to interactions of different amino acids. *F*, the interaction between Arg849 and Thr885 quantified using RDFs. The RDF is constructed between the hydroxyl oxygen of the Thr885 side chain and the terminal carbon atom of the Arg849 side chain. All RDFs for the all-atom simulations are collected from the last 200 ns of three simulation replicas each for DOPS and PI(3,4,5)P_3_. DOPS, 1,2-dioleoyl-*sn*-glycero-3-phospho-l-serine; PI(3,5)P_2_, phosphatidylinositol 3,5-bisphosphate; PI(4,5)P_2_, phosphatidylinositol 4,5-bisphosphate.

To investigate whether lipids can spontaneously approach the protein's lipid-binding site, we first performed coarse-grained (CG) MD simulations with the protein embedded in a membrane containing phosphatidylcholine, phosphatidylethanolamine, PS, and Bis(monoacylglycerol)phosphate found in the endosome membrane. In addition, we introduced either phosphatidylinositol 3,5-bisphosphate, phosphatidylinositol 4,5-bisphosphate, or phosphatidylinositol 3,4,5-trisphosphate (PI(3,4,5)P_3_) into both membrane leaflets (see Table S2 for a detailed composition of the membranes). In most replica simulations, a PIP lipid consistently approached and bound to the lipid-binding site (Figs. S5 and 6*A*). Occasionally, other anionic lipids, such as PS and Bis(monoacylglycerol)phosphate, also interacted with the site, but the radial distribution functions in Figure 6*B* suggest that PIP lipids exhibited the strongest binding.

To determine whether PI(3,4,5)P_3_ was favored over the diphosphate variants, we conducted 10 replicas of a system where PI(3,4,5)P_3_ and phosphatidylinositol 3,5-bisphosphate were present at equal concentrations in the outer leaflet. However, our results did not indicate a clear preference between the two lipids over the 10 replicas (Fig. 6*C*). Overall, these findings suggest that while the binding site has a strong preference for PIP lipids, the protein exhibits a significant degree of promiscuity in lipid selection. These data are in good agreement with the experimental ATPase activities shown in Figure 5.

To gain molecular insights into the interaction between bound PI(3,4,5)P_3_ lipid and the protein, we back-mapped a PI(3,4,5)P_3_-bound CG configuration into an all-atom representation (Fig. S5). We then performed three replicas of 500 ns all-atom simulations with the PI(3,4,5)P_3_-bound protein embedded in a lipid membrane. While we initially expected the phosphate groups of either the sugar headgroup or the lipid backbone to directly interact with the cationic side chain of Arg849, no direct interactions with Arg849 were observed. Instead, the sugar headgroup formed strong hydrogen bonds and van der Waals contacts with residues Thr104, Pro108, Asn341, Ile345, and Tyr872 (Fig. 6, *D* and *E*). Although Arg849 did not directly contact the phosphates, its cationic environment likely contributed to anchoring the PI(3,4,5)P_3_ lipid within the binding site.

To further explore lipid-binding specificity, we replaced PI(3,4,5)P_3_ with a PS lipid and relaunched the all-atom simulations. Due to its smaller headgroup, PS penetrates much deeper into the binding pocket compared with PI(3,4,5)P_3_ and interacts with Arg849 (Fig. 6, *D* and *E*). The PS headgroup also formed hydrogen bonds with Asn341, Ser346, and Thr882. The Asn341 located at the unwound portion of TM4 in ATP9A is conserved in the PS-specific ATP11C flippase, and its corresponding Asn352 in ATP11C is vital for the PS-dependent ATPase activity and its transport (9, 10). These observations suggest that the headgroups of various phospholipids are recognized in a manner suitable for each at the large binding pocket of ATP9A.

Notably, we observed a stable interaction between Arg849 and Thr885 throughout the simulation (Fig. 6*F*), which is consistent with the pivotal role of Arg849 in luminal gating observed in the cryo-EM structure (Fig. 3).

### Differences between canonical gating and ATP9A TM6 gating

The here-reported structural analysis of conformational changes in ATP9A allowed us to formulate a gating mechanism uniquely found in ATP9A hitherto unobserved in other P-type ATPases. The relationship between transport substrates and gate opening/closing is well understood, especially for P2-type ATPases (28, 29) as well as in ATP11C (10), as the “canonical” gating mechanism. In all cases for other P-type ATPases so far, 10 TM helices are divided into a transport domain (TM1–TM6) that binds and transports substrate and a structural support domain that works as an anchor (TM7–TM10) (30). Especially in human P4-ATPases, including ATP11C (10) and ATP8B1 (2), outward gating is achieved by TM1 and TM2, and other TM helices barely move during the transport cycle. Starting from the inward-facing E1P state, where the P domain is segregated from the A domain and the outer gate is tightly closed, the outer gate opening is triggered by the E2P formation in which the P domain aspartylphosphate is covered by the conserved DGES/T (TGES for P2-ATPases) loop in the A domain (Fig. 1*B*, *inset*). This transition from E1P to E2P requires a large movement of the A domain, and since the A domain connects TM1 and TM2, the outer gate opening is achieved by the movement of these two helices, allowing the PS headgroup to reach the binding site. The binding of the PS headgroup itself, in turn, induces outer gate closure by making hydrogen bonds with some hydrophilic residues in the TM1–2 loop; hence, the PS headgroup becomes occluded in the binding site. The TM1–2 movement is transmitted to the A domain, and the DGES/T loop moves aside from the phosphorylation site, which allows water access to hydrolyze aspartylphosphate at the P domain (10). Given that the above sequence of conformational changes is coupled with the phosphorylation and dephosphorylation reaction, it is possible to say that the canonical gating is an energy-driven process. In contrast, the opening/closure of the outward gate in ATP9A is observed in the same E2P state. We could not find any significant differences in the relative orientation of the cytoplasmic domains or around the phosphate analog in the P domain in ATP9A open/closed states (Figs. 1 and 2). These observations suggest that the TM6 gating is a spontaneous and stochastic process in the E2P state, essentially not requiring any energy input. Despite our attempts to analyze the structure in the presence of AMPPCP or AlF, all obtained structures are in the E2P state, and thus, we could not obtain E1P and E2-P_i_ states so far (Fig. S4). However, given that very slow but significant phospholipid-dependent ATPase activity was detected (Fig. 5), it is likely that ATP9A undergoes a canonical conformational change according to the Post-Albers type reaction scheme. Due to the strong preference for the E2P state (31), the accumulation of other reaction intermediates (E1P or E2-P_i_ states), which are populated in the native transport cycle, may be hindered in ATP9A.

### Physiological function of ATP9A

The stable E2P conformation observed in our *in vitro* structural analysis is consistent with the very slow turnover detected in our ATPase activity measurements (Fig. 5). Assuming 100% purity of the ATP9A sample with a molecular weight of 90 kDa, the maximum specific activity of approximately 0.4 μmol/mg/h corresponds to a turnover number of 0.01/s, that is, one molecule of ATP is hydrolyzed every 100 s, which is more than 1000 times slower than that of ATP11C purified in similar conditions (10) and 60 times slower than that of Neo1 (11). One question that emerges is whether this slow turnover of ATP9A itself has any impact on the physiological functions attributed to the lipid transport in living cells. As the activity measurement is performed using delipidated purified protein in the detergent micelle, we cannot exclude the possibility that an environment different from that of lipid bilayers, including annular lipid and rigid membrane structure itself, is responsible for the reduced turnover rate, which is frequently observed in other P-type ATPases (32, 33, 34). Very recently, Graham *et al.* (35)demonstrated PIP exposure in ATP9A KO cells, suggesting that ATP9A acts as an active flippase within a biological membrane. Accordingly, the extremely slow turnover observed in our purified, detergent-solubilized ATP9A preparation is highly likely attributable to the absence of bulk lipids and the rigid membrane scaffold they form. The crystal structure analysis of sarcoplasmic/endoplasmic reticulum Ca^2+^-ATPase in a lipid environment revealed that the tilting of the molecule relative to the membrane plane contributes to maintaining the high-energy state of reaction intermediates (36). In contrast, within detergent micelles, even if a certain amount of phospholipid is present, the flexible structure of detergent micelles would not allow the ATP9A molecule to tilt in a similar manner. Therefore, our data, a large lipid binding pocket that allows PIP1 binding (Figs. 4 and 6), and significant activation of ATPase activity in the presence of PIPs (Fig. 5), qualitatively consistent with the PIP flipping activity in the living cell membrane. Although ATP9A and ATP9B are believed to work as a monomer (2), a recent study suggests a possibility of homodimer or heterodimer formation (37). The existence of an unknown auxiliary subunit also could not be excluded.

Alternatively, previous studies have demonstrated that ATP9A was shown to be required for the recycling pathway from the endosome to the plasma membrane (15) and exosome release (14), suggesting that ATP9A is involved in cellular membrane traffic. It has recently been shown that ATP9A pathogenic mutants lead to neurodevelopmental disorder and synaptic dysfunction through the regulation of the small G-proteins Rab5 and Rab11 (16). As suggested previously (38), an unusually slow turnover and the accumulation of a stable E2P conformation observed in ATP9A lead us to speculate that the physiological role of ATP9A in the cell may be as a scaffold for proteins involved in membrane trafficking, rather than its phospholipid flippase activity. Analysis of phospholipid binding–deficient mutants and/or direct observation of the complex structure will be required to test this hypothesis.

Our ATPase measurement shows that, despite low substrate specificity, the activation by the addition of PIPs is significantly higher than others, which is consistent with the results obtained by MD simulations. However, PIPs are important for the intracellular signaling and are mainly distributed in the inner leaflet. This implies that the binding of PIPs from the outer leaflet is unlikely to occur in a physiological environment. Nevertheless, a recent report suggests the presence of PIPs on the cell surface using a PIP-specific antibody (39), and these appear to be important for the adhesion and the establishment of cell polarity. The flipping activity of PIPs by ATP9A at the endosomal membrane where it is localized may be to avoid unnecessary exposure of PIPs to the outer leaflet.

## Conclusion

In this article, through structure–function analysis and MD simulations of monomeric ATP9A, we showed the mechanistic rationale of outward phospholipid gating occurring in the E2P state and suggested its potential substrates. The novel gating mechanism, never seen in any other P-type ATPases, may be acquired to form a large phospholipid-binding cavity uniquely found in ATP9A. A series of conformational changes required for this gating would not be possible with other flippases in which most TM helices, including TM6, are tightly bound to CDC50 protein, and thus would only be possible in the monomeric ATP9A. Conversely, this may imply that CDC50 protein is required for other flippases to gain finer phospholipid specificity by tightening its binding cavity suitable for their specific substrates.

## Experimental procedures

### Expression and purification

Procedures for protein expression and purification are essentially the same as those reported previously (10, 17, 18). Briefly, a hexa-histidine tag and the enhanced GFP were inserted in the amino-terminal side of Pro38 of the human ATP9A and followed by a human rhinovirus 3C (HRV-3C) protease recognition sequence and subcloned into a hand-made vector (24). We designed a mutant lacking the N-terminal region because AlphaFold2 predicted it to be an intrinsically disordered region, to prevent intracellular cleavage during expression, and to improve stability of the protein during structural analysis. The ATP9A was expressed using baculovirus-mediated transduction of mammalian human embryonic kidney 293S GnT1^-^ cells (BacMam) (17) purchased from the American Type Culture Collection. Cells expressing ATP9A were directly solubilized with 1% lauryl maltose neopentyl glycol (LMNG) in the presence of 40 mM Mes–Tris (pH 6.5), 5% glycerol, 5 mM dithiothreitol, 200 mM NaCl, 5 mM MgCl_2_, in the presence of 1 mM BeSO_4_ and 3 mM NaF (for BeF condition), 1 mM AlCl_3_ and 4 mM NaF (for AlF), or 5 mM ATP (for AMPPCP condition or activity measurement) on ice for 20 min. After removal of insoluble material by ultracentrifugation, the supernatant was mixed with anti-GFP nanobody resin (40) at 4 °C for 3 h, which was followed by washing with buffer containing 40 mM Mes–Tris (pH 6.5), 5% glycerol, 200 mM NaCl, 1 mM MgCl_2_, and 0.06% glyco-diosgenin, in the presence or absence of phosphate analogs depending on the conditions. After the addition of HRV-3C protease, anti-GFP nanobody was incubated at 4 °C overnight. Digested peptide fragments containing enhanced GFP and HRV-3C protease were removed by passing the fractions through a nickel–nitrilotriacetic acid resin (Qiagen). Flow-through fractions were concentrated and subjected to further purification with size-exclusion column chromatography using a Superose 6 Increase column equilibrated in buffer comprising 20 mM Mes–Tris (pH 6.5), 1% glycerol, 200 mM NaCl, 5 mM MgCl_2,_ and 0.06% glyco-diosgenin with or without phosphate analogs. Peak fractions were collected and concentrated to 8 mg/ml and subjected to cryo-EM analysis. We added 5 mM AMPPCP as a final concentration only for the AMPPCP sample. For the ATPase measurement, anti-GFP nanobody resin was washed with a buffer containing 0.06% LMNG and 0.1 mM ATP. After overnight treatment with HRV-3C protease and following nickel–nitrilotriacetic acid, the sample was concentrated to 4 mg/ml and subjected to the ATPase measurement as described later.

### Cryo-EM and structure analysis

Preparation of the sample and cryo-EM grid was done according to the previous report (10). The purified protein samples (at 8 mg/ml) were applied to a freshly glow-discharged Quantifoil holey carbon grid (R1.2/1.3, Cu/Rh, 200 mesh), using a Vitrobot Mark IV (FEI) at 4 °C with a blotting time of 4 s under 99% humidity, and the grids were then plunge-frozen in liquid ethane. Prepared grids were transferred to a CRYO ARM 300 microscope (JEOL), running at 300 kV and equipped with a Gatan K3 Summit direct electron detector in the electron counting mode. Imaging was performed at a nominal magnification of 60,000×, corresponding to a calibrated pixel size of 0.752 Å/pix (SPring-8 EM01CT). Each movie was recorded in a correlated-double sampling mode for 2.6 s and subdivided into 60 frames. The electron flux was set to 8.46 e^−^/pix/s at the detector, resulting in an accumulated exposure of 60 e^−^/Å^2^ at the specimen. The data were automatically acquired by the image shift method using SerialEM software (41), with a target defocus range of −1.4 to −1.6 μm. The dose-fractionated movies were subjected to beam-induced motion correction, using Relion 3.1 (42), and the contrast transfer function (CTF) parameters were estimated using patch CTF estimation in cryoSPARC v4 (Structura Biotechnology Inc. Toronto, Canada) (43).

For each dataset, particles were initially picked by blob picker using cryoSPARC and extracted with downsampling to a pixel size of 3.24 Å/pix. These particles were subjected to several rounds of 2D classifications. The 2D classes that show clear secondary structures were then subjected to *ab initio* reconstruction in three models and refined by nonuniform refinement. The best class was then re-extracted with a pixel size of 0.752 Å/pix and subjected to nonuniform refinement with on-the-fly per-particle defocus refinement and beam-tilt refinement in cryoSPARC. The density for several helices, including that of TM6, was slightly weaker compared with the rest of the molecule. To help visualize any potential variable conformations, we employed 3D variability analysis (three orthogonal modes) on the previous nonuniform refinement reconstruction, using a soft mask encompassing the protein molecule (excluding the detergent micelle). The subsequent results were outputted in simple mode to produce a linear movie of volumes (using 20 frames, Movie S1), which was displayed as an oscillating volume series in ChimeraX (44). The presence of two potentially variable conformations of TM6 and TM9 was clearly visible at this stage. To separate these conformations, the particle set was then transferred to RELION to perform masked 3D classification (with no alignment), where two classes (corresponding to the open and closed states) and their respective particle sets were separately subjected to Bayesian polishing (45). Polished particles were reimported to cryoSPARC, and nonuniform refinement (with on-the-fly CTF refinement) was performed separately on each class, with the closed state reaching 2.31 Å and the open state reaching 2.18 Å resolution. Resolutions of the analyzed maps were defined according to the gold standard Fourier shell correlation = 0.143 criterion (46). The local resolution and angular distributions for each structure were estimated by cryoSPARC. The model of E2P open form was manually built in Coot 0.9.8 (47) using a homology model generated by SWISS-MODEL server (48), based on the cryo-EM structure of ATP11C (Protein Data Bank ID: 7BSU) as a template structure (10). PHENIX (49) was used for refinement (real space) of the atomic model into the density map.

### ATPase assay

The affinity-purified ATP9A sample with 0.1 mM ATP and 0.06% LMNG was used for the ATPase measurement as described previously (9, 10). Briefly, samples were diluted to 0.4 mg/ml in buffer containing 40 mM Pipes–Tris (pH 7.0), 2 mM MgCl_2_, and 2 mM ATP in the absence and presence of 20 μM various phospholipids dissolved in 2% C_12_E_8_. The final concentration of C_12_E_8_ is adjusted to 0.02%. Reactions were initiated by incubating the fractions at 37 °C using a thermal cycler and maintained for 2 h. Reactions were terminated by adding 1% SDS and 2 M HCl, and the amount of inorganic phosphate released was determined colorimetrically (50) using a microplate reader (Thermo). The specific ATPase activity was calculated by subtracting the activity in the sample containing 1 mM BeSO_4_ and 3 mM NaF.

### Simulations

The open conformation of the P4-ATP9A flippase model was used as the initial coordinates for the MD simulation. Subsequently, the missing regions of the protein were modeled using the MODELLER suite (51), incorporating the phosphorylated aspartic acid and an Mg^2+^ ion.

### CG MD simulations

Initially, the atomistic protein structure was converted into a low-resolution bead model using the *martinize* program (52) with the MARTINI3 force field (53) and a strong elastic network (54) and a force constant of 500 kJ mol^−1^ nm^−2^. The Define Secondary Structure of Proteins program (55) was used to define the secondary structure, atom types, and bonded interactions of the protein. A force constant of 500 kJ mol^−1^ nm^−2^ was applied to the protein backbone atoms.

The *insane.py* script (56) was used to center and embed the protein within homogeneous and heterogeneous membrane bilayer models (Table S2) with lipids randomly positioned around the protein. The membrane composition of each system is described in Table S2. The composition of the inner and outer leaflets was kept different.

The periodic box dimensions were set to 250 × 250 × 180 Å. The system was solvated with water beads and neutralized with NaCl at a concentration of 0.15 M.

Subsequently, the systems underwent 5000 steps of energy minimization using the steepest descent algorithm, followed by equilibration for 10 ns with periodic boundary conditions applied in all directions. During equilibration, pressure was set with the Berendsen barostat using semi-isotropic pressure coupling (57). Finally, production runs were conducted for 10 μs using the Parrinello–Rahman barostat (58), with a coupling constant of 12 ps and a compressibility of 3 × 10^−4^ bar for pressure control. The velocity-rescale thermostat was used for all simulations to set the temperature to 310 K (59). The rest of the simulation protocol was standard, and the same was reported in our earlier work (60).

### Backmapping to atomistic models

A representative snapshot was extracted from the CG MD trajectories and converted to all-atom resolution using the *backmap.py* program (6) (Fig. S2) with the CHARMM36 force field (61), following the automated protocol available in CHARMM-GUI (62).

### All-atom simulations

The systems underwent 5000 steps of energy minimization using the steepest descent algorithm, followed by six default equilibration stages that gradually relaxed the restraint forces on the protein backbone and the bound lipid molecule. Prior to the production run, the backbone atoms of the protein were restrained and subjected to a 100 ns run. Finally, unrestrained production runs were conducted for 500 ns per system. The temperature was maintained at 310 K using the V-scale thermostat (59), whereas the pressure was controlled using the Parrinello–Rahman barostat (58) with 2 fs time integration. The rest of the simulation protocol was similar to our previous work (63). All simulations were performed using GROMACS, version 2024.3 (64). Postprocessing of all trajectories was performed using GROMACS and in-house Bash and Python scripts. Graphical rendering was performed with VMD (65) and PyMOL (https://pymol.org).

## Data availability

The data that support this study are available from the corresponding author upon reasonable request. The data needed to evaluate the conclusion of the article are either in the article or in the supporting information. The following atomic models and a cryo-EM map have been deposited in the Protein Data Bank (https://www.rcsb.org/) and the Electron Microscopy Data Bank, respectively.

9VDK: Cryo-EM structure of human ATP9A in BeF-bound E2P state open form (https://doi.org/10.2210/pdb9vdk/pdb)

EMD-64986: Cryo-EM structure of human ATP9A in BeF-bound E2P state open form (https://www.ebi.ac.uk/pdbe/entry/emdb/EMD-64986)

9VDL: Cryo-EM structure of human ATP9A in BeF-bound E2P state closed form (https://doi.org/10.2210/pdb9vdl/pdb)

EMD-64987: Cryo-EM structure of human ATP9A in BeF-bound E2P state closed form (https://www.ebi.ac.uk/pdbe/entry/emdb/EMD-64987)

9VDM: Cryo-EM structure of human ATP9A (AMPPCP) E2P state open form (https://doi.org/10.2210/pdb9vdm/pdb)

EMD-64988: Cryo-EM structure of human ATP9A (AMPPCP) E2P state open form (https://www.ebi.ac.uk/pdbe/entry/emdb/EMD-64988)

9VDN: Cryo-EM structure of human ATP9A (AlF) E2P state open form (https://doi.org/10.2210/pdb9vdn/pdb)

EMD-64989: Cryo-EM structure of human ATP9A (AlF) E2P state open form (https://www.ebi.ac.uk/pdbe/entry/emdb/EMD-64989).

## Supporting information

This article contains supporting information.

## Conflict of interest

The authors declare that they have no conflicts of interest with the contents of this article.
