# Supplementary material for: A unique gating mechanism revealed by the cryo-EM structure of monomeric ATP9A flippase
Source: J Biol Chem. 2025 Aug 26;301(10):110631. doi: 10.1016/j.jbc.2025.110631 (PMC12494553; doi:10.1016/j.jbc.2025.110631)
Supplement: Supporting Information [file mmc2.pdf]

Supporting Information for

A unique gating mechanism revealed by the cryo-EM structure of monomeric ATP9A flippase

Kazuhiro Abe[1*], Parthiban Marimuthu[2], Qian Yuheng[1,3], Chai Gopalasingam[1,4], Christoph Gerle[4], Hideki Shigematsu[5], Kotaro Tanaka[3], Himanshu Khandelia[6]

[1]Department of Chemistry, Faculty of Science, Hokkaido University, Japan

[2]Pharmaceutical science laboratory (PSL – Pharmacy) and Structural Bioinformatics Laboratory (SBL – Biochemistry), Faculty of Science and Engineering, Åbo Akademi University, FI-20520 Turku, Finland

[3]Graduate School of Pharmaceutical Sciences, Nagoya University, Nagoya, Japan

[4]RIKEN SPring-8 Center, Kouto, Sayo-gun, Hyogo 679-5148, Japan

[5]Japan Synchrotron Radiation Research Institute (JASRI), SPring-8, 1-1-1 Kouto, Sayo, Hyogo 679-5148, Japan

[6]Department of Physics Chemistry and Pharmacy, University of Southern Denmark, Odense, Denmark

*Correspondence should be addressed: kabe@sci.hokudai.ac.jp

Running title: Cryo-EM structures of a monomeric ATP9A flippase

Keywords: ATPase, cryo-electron microscopy, lipid transport, membrane protein, phospholipid, transporter

Table S1| **Statistics of the structural analysis**

	Cryo-EM structure of human ATP9A in BeF-bound E2P state open form	Cryo-EM structure of human ATP9A in BeF-bound E2P state closed form	Cryo-EM structure of human ATP9A (AMPPCP) E2P state open form	Cryo-EM structure of human ATP9A (AlF) E2P state open form
PDB	9VDK	9VDL	9VDM	9VDN
EMDB	EMD-64986	EMD-64987	EMD-64988	EMD-64989
**Data collection and processing**				
Magnification	60,000			
Voltage (kV)	300(CRYO ARM 300)			
Electron exposure ($e^-/Å^2$)	60			
Defocus range (μm)	0.8-1.8			
Pixel size (Å)	0.752			
Symmetry imposed	*C1*			
Initial particle images (no.)	5,577,204		3,666,132	4,779,021
Final particle images (no.)	126,825	84,464	59,377	101,903
Map resolution (Å)	2.18	2.31	3.01	2.61
FSC threshold	0.143			
**Refinement**				
Initial model used (PDB code)	7bsu	9VDK	9VDK	9VDK
Model resolution (Å)	2.3	2.5	3.2	2.8
FSC threshold	0.5			
Map sharpning *B* factor ($Å^2$)	-45.7	-46.6	-82.3	-63.5
Model composition				
Non-hydrogen atoms	8,429	8,242	7,980	8,188
Protein residues	997	1,000	993	997
Ligands	Mg^{2+}, BFD, 3PCW, CLR, P5S, 298water	Mg^{2+}, BFD, 270water	Mg^{2+}, CLR, P5S	Mg^{2+}, ALF, 4PCW, CLR, P5S
B factors ($Å^2$)				
Protein	45.74	59.68	87.85	71.00
Ligand	55.30	44.64	103.83	80.46
Water	43.84	54.27	-	-
R.m.s. deviations				
Bond length (Å)	0.003	0.004	0.003	0.002
Bond angles (º)	0.992	0.999	0.608	0.731
Validation				
Mol Probity score	1.53	1.83	1.81	1.73
Clashscore	6.77	7.05	8.21	4.59
Poor rotamers (%)	1.59	3.84	2.62	4.19
Ramachandran plot				
Favored (%)	98.69	98.59	97.78	97.89
Allowed (%)	1.31	1.41	2.12	2.01
Disallowed (%)	0.00	0.00	0.10	0.10

Table S2| **Lipid compositions for simulations**

No difference is there is PS in the lower leaflet in all-atom simulations

No PIP binds if PIP is only in the inner leaflet

One line about importance of cholesterol

Symmetric CG systems: Same composition of lower and upper leaflets in terms of PS, BMP and PIP lipids. (5 x 10 μs)

**List of Simulations**	
**CG Asymmetric systems**	**Duration (μs)**
DOPC:40, DOPE:20, PIP345:05, BMP:05, CHOL:30 (U)<br>DOPC:40, DOPE:15, DOPS:05, PIP345:05, BMP:05, CHOL:30 (L)	5 x 10
DOPC:40, DOPE:20, PIP35:05, BMP:05, CHOL:30 (U)<br>DOPC:40, DOPE:15, DOPS:05, PIP35:05, BMP:05, CHOL:30 (L)	5 x 10
DOPC:40, DOPE:20, PIP45:05, BMP:05, CHOL:30 (U)<br>DOPC:40, DOPE:15, DOPS:05, PIP45:05, BMP:05, CHOL:30 (L)	5 x 10
DOPC:40, DOPE:20, PIP345:0.25, PIP35:0.25, BMP:05, CHOL:30 (U)<br>DOPC:40, DOPE:15, DOPS:05, PIP345:05, BMP:05, CHOL:30 (L)	10 x 10
**All-atom simulations**	
DOPC:70, CHOL:30 (U) **(with bound PIP345)**<br>DOPC:60, DOPS:10, CHOL:30 (L)	3 x 0.5
DOPC:70, CHOL:30 (U) **(with bound PS)**<br>DOPC:70, DOPS:10, CHOL:30 (L)	3 x 0.5

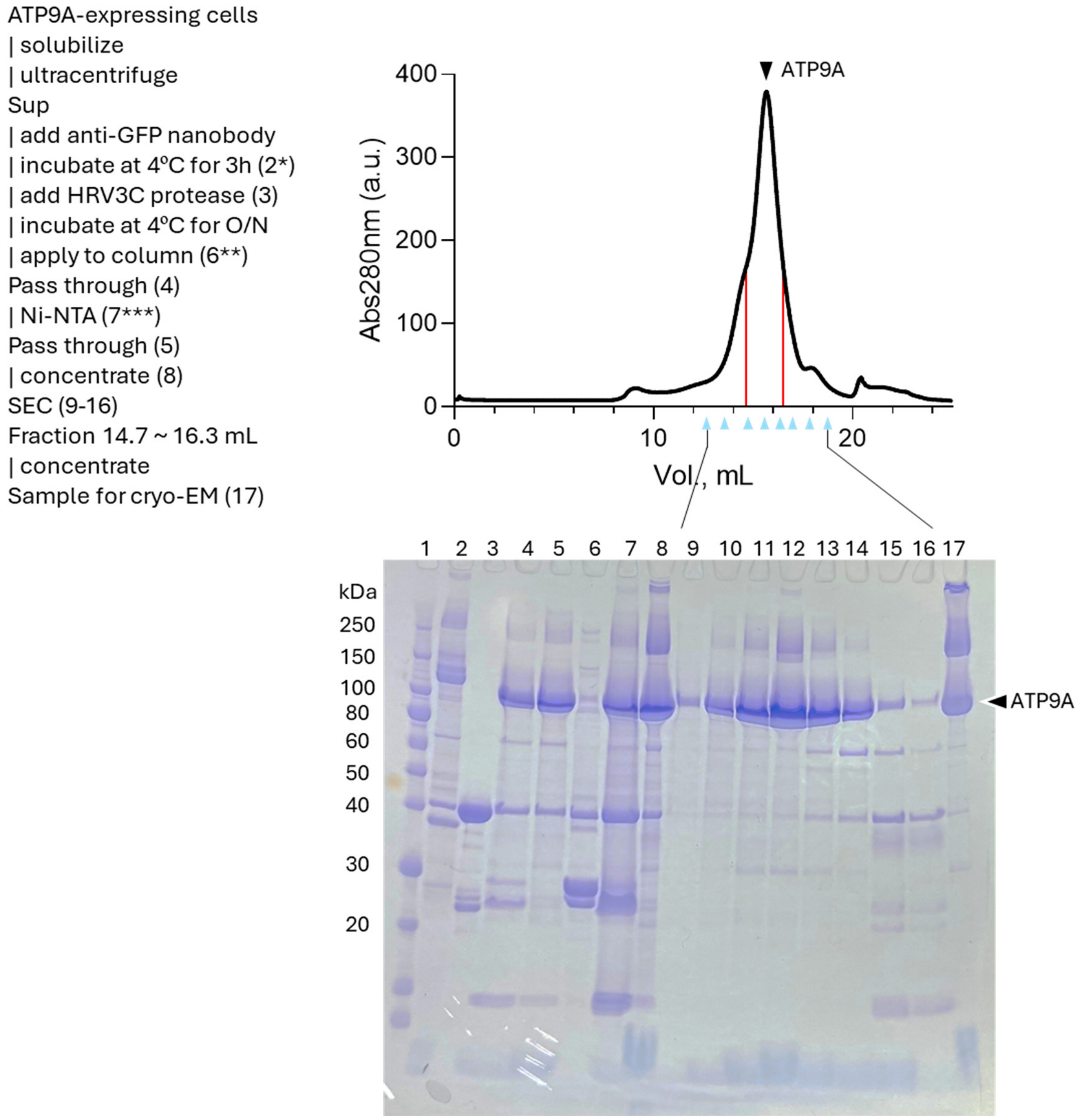

Fig. S1| **Purification of ATP9A**

A brief workflow of ATP9A purification (left), elution profile of size-exclusion chromatography (right) and SDS-PAGE analysis (lower right). Numbers in parentheses correspond to the lane number of the SDS-PAGE gel. Fractions between red lines are collected and used for the cryo-EM analysis. Light blue arrowheads indicate fractions analyzed by SDS-PAGE. *A sample before HRV3C protease cleavage. **Elution with 0.1 M glycine/HCl (pH 2.0) to identify proteins remaining on the anti-GFP nanobody resin. ***Elution with 0.5 M imidazole/HCl (pH 8.0) to identify proteins trapped on Ni-NTA resin.

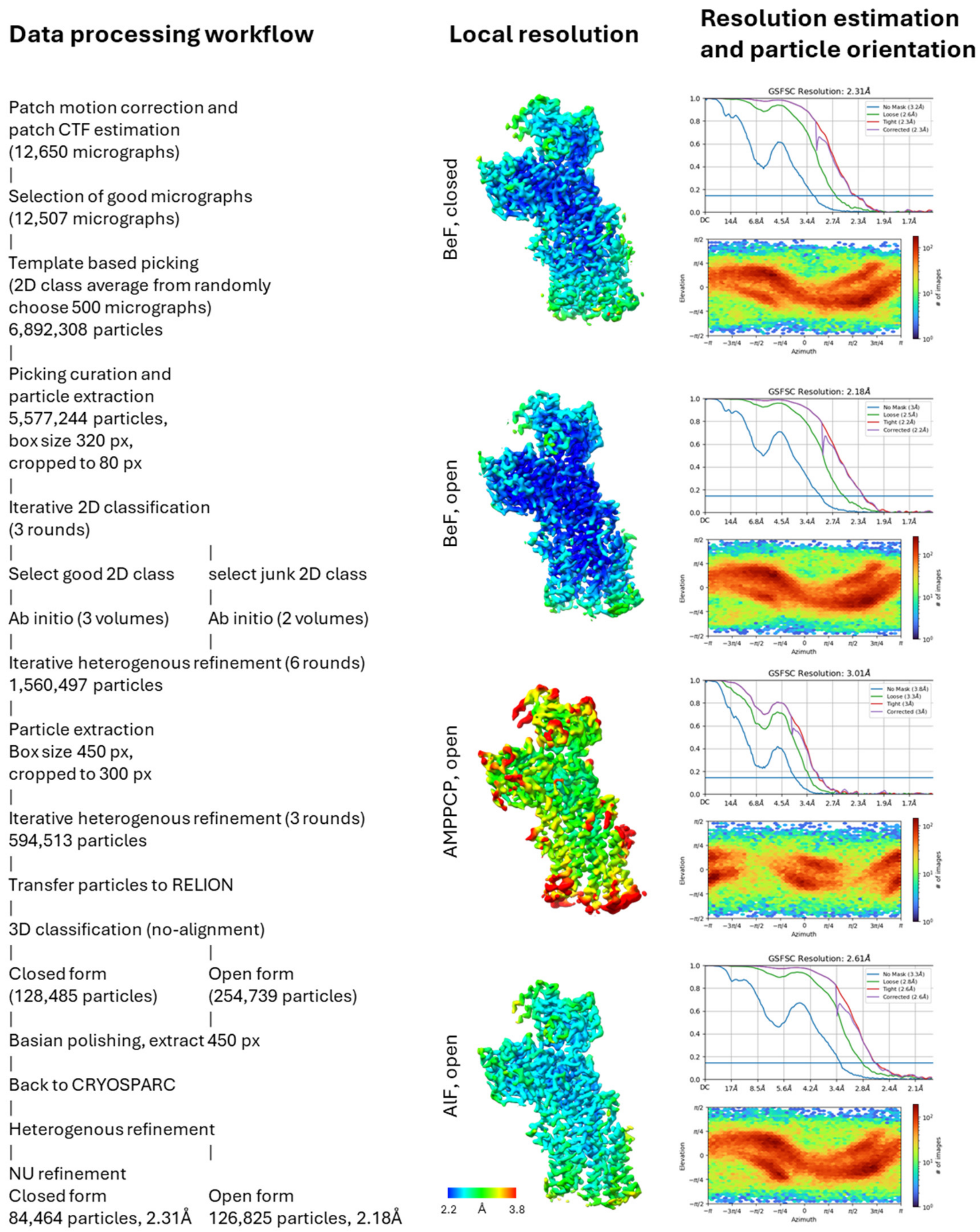

Fig. S2| **Cryo-EM analysis**

The cryo-EM data processing workflow (left), local resolution calculated by cryoSPARC (center, Scale for the local resolution is indicated in the figure), Fourier Shell Correlation (FCS) plots used for resolution estimation and angular distribution plots of the particles included in the 3D reconstruction (right) are shown for the indicated dataset.

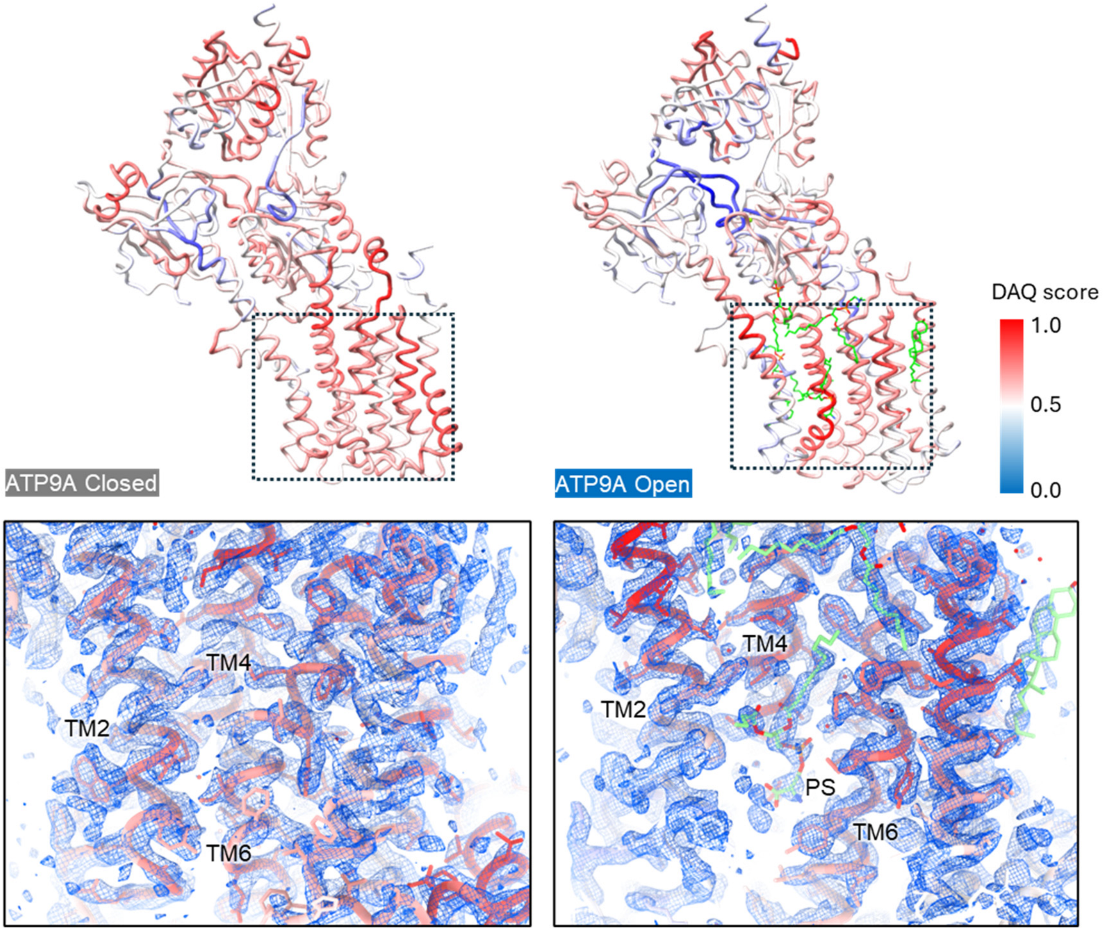

Fig. S3| **Cryo-EM density maps and DAQ-Scores for closed and open forms**

Upper panels show ribbon models for ATP9A BeF-bound E2P state closed form (left) and open form (right). Close-up view of sharpened EM maps (blue mesh) with superimposed models are shown in the lower panels. Models are colored with their calculated DAQ score (22), where higher value shows more reliable modeling. Lipid molecules are shown as green sticks.

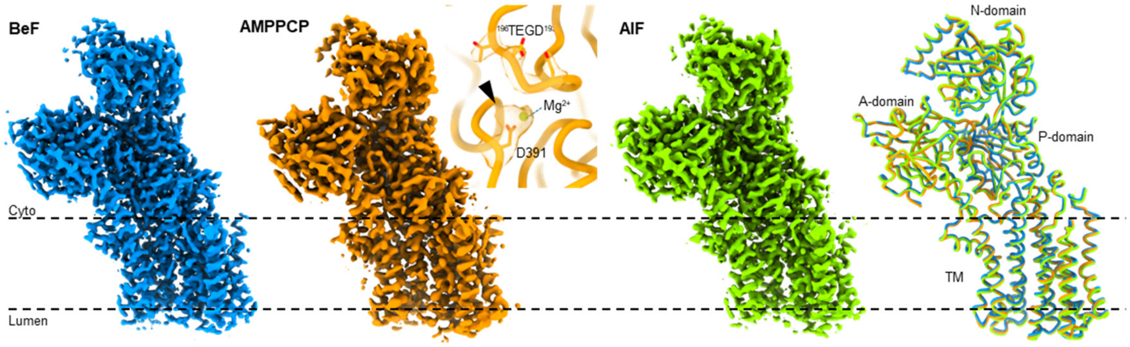

Fig. S4| **Comparison of open form structures from BeF, AMPPCP and AIF samples**

Sharpened maps of open form obtained from indicated samples are shown. Close-up of phosphorylation site (Asp391) from AMPPCP sample is shown in inset. An arrowhead points to the excess EM density found at the phosphorylation site in AMPPCP sample, indicating that the catalytic Asp391 remains to be phosphorylated during purification step. Superimposition of amino acid models is shown in the right panel.

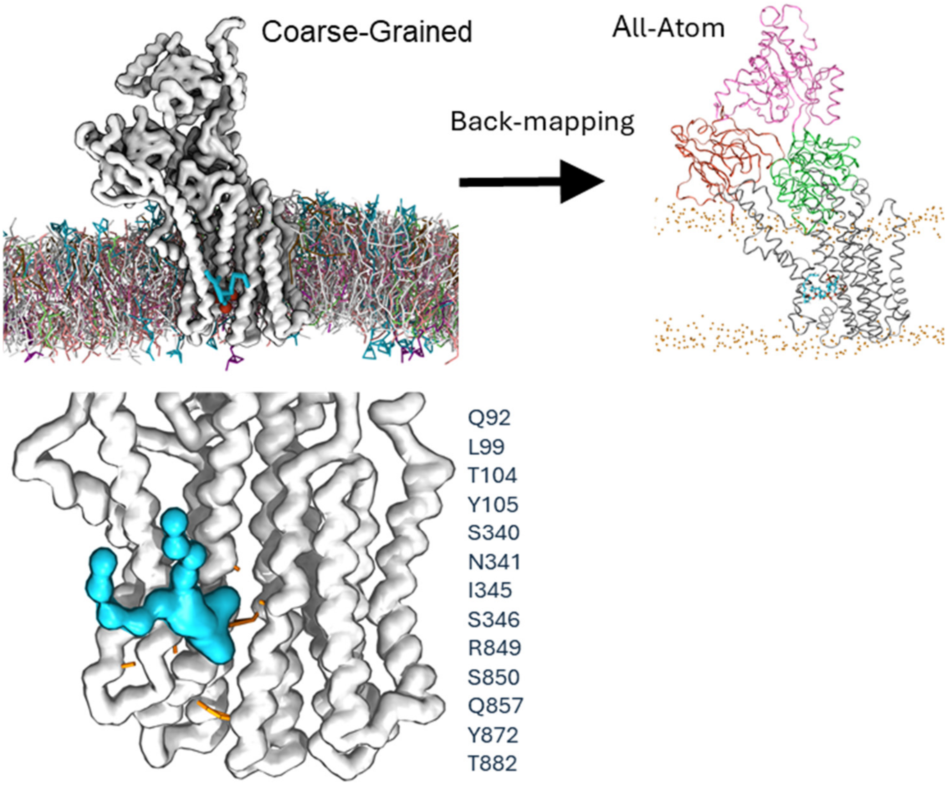

Fig. S5| **Back mapping into the atomistic model**

The backmapping of a PI(3,4,5)P_3 bound coarse-grained structure into an all-atom structure. The lipid is shown in blue and the binding site residues are shown in yellow in the coarse-grained structure.

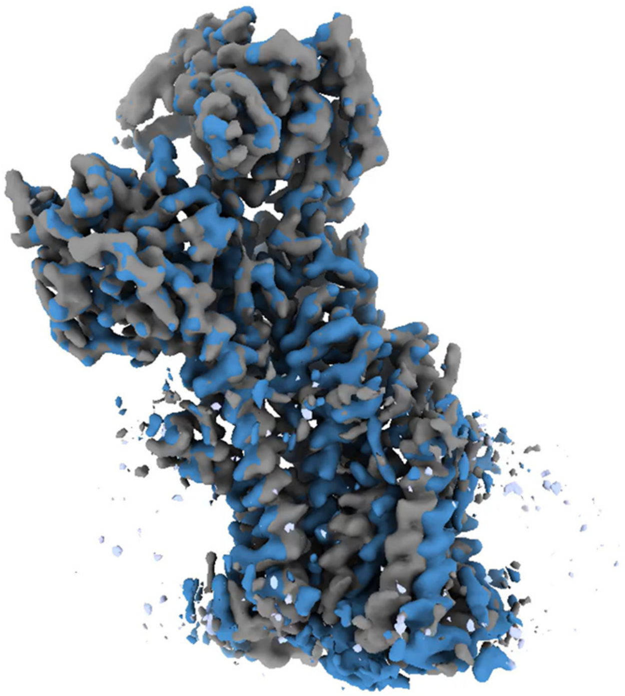

Movie S1| **3D variability analysis**
